# Molecular detection and genetic diversity of *Rickettsia* spp. in pet dogs and their infesting ticks in Harbin, northeastern China

**DOI:** 10.1186/s12917-021-02823-y

**Published:** 2021-03-07

**Authors:** Jian-Wei Shao, Xin-Yan Yao, Xu-Dong Song, Wen-Jun Li, Hui-Lan Huang, Shu-Jian Huang, Xue-Lian Zhang

**Affiliations:** 1 Key Laboratory for Preventive Research of Emerging Animal Diseases, Foshan University, 528231 Foshan, Guangdong China; 2 College of Life Science and Engineering, Foshan University, 528231 Foshan, Guangdong China; 3 Dr.Song’s Clinic, 150086 Harbin, Heilongjiang China

**Keywords:** *Rickettsia* spp., Pet dogs, Molecular epidemiology, Northeastern China, One health

## Abstract

**Background:**

Pet dogs are important companion animals that share the environment within households, and play an important role in local community life. In addition, pet dogs also are reservoirs of zoonotic agents, including *Rickettsia* spp., thus increasing the risk of rickettsial infections in humans. It’s meaningful to investigate the epidemiology of rickettsial agents in pet dogs, and make contribute to the surveillance of rickettsioses in human in China.

**Results:**

In this study, a total of 496 pet dogs’ blood samples and 343 ticks infested in pet dogs were collected, and the presence and prevalence of *Rickettsia* were determined by amplifying the partial *gltA* and *17-kDa* genes, with an overall positive rate of 8.1 % in blood samples and 14.0 % in tick samples. In addition, the *rrs*, *gltA*, *groEL*, and *ompA* genes of rickettsial were also recovered to determine the species of *Rickettsia* detected furtherly. Sequencing blast and phylogenetic analyses revealed the presence of three human pathogenic *Rickettsia* species (*Rickettsia raoultii*, *Candidatus* Rickettsia tarasevichiae and *Rickettsia felis*) in samples associated with pet dogs. Moreover, all the sequences of *Rickettsia* that we obtained presented close relationship with others available in GenBank, and *Rickettsia raoultii* was the most predominant *Rickettsia* species infected in pet dogs’ blood samples or in tick samples.

**Conclusions:**

This study provides the molecular epidemiology data about the Rickettsia spp. infection associated with pet dogs in urban areas of Harbin city. Three rickettisae species pathogenic to humans were identified from pet dogs’ blood and the infested ticks in urban areas of Harbin city. Considering the intimate relationship between human and pets, these results indicate the potential transmission risk of human rickettisal infections from pet dogs through ectoparasites, and also highlighting that more attention should be paid to rickettsial infection in pet dogs and the infested ticks from the “One health” perspective.

## Background

Rickettsioses, which caused by *Rickettsia* spp., are important emerging vector-borne diseases in humans [1], and some have been reported to infect dogs [2, 3]. Genus *Rickettsia* (family Rickettsiaceae, order Rickettsiales) are a large group of Gram-negative obligate intracellular prokaryotic microbes [4]. They are widely distributed throughout the world, and maintained and transmitted by vector arthropods such as ticks, fleas, mites, lice and mosquitos [5]. In recent years, an increasing number of novel *Rickettsia* species have been described, and human diseases caused by known or novel *Rickettsia* species have been continuously reported, such as *Rickettsia sibirica subsp. sibirica*, *R. raoultii*, *R. subsp.* XY99 and *Candidatus* Rickettsia tarasevichiae in China [6–9], and *R. monacensis* in Europe and South Korea [10, 11].

In the past 30 years, at least twelve valid *Rickettsia* species, including *R. heilongjiangensis*, *R. raoultii*, *R. rickettsia*, *R. conorii*, *R. aeschlimannii*, *R. massiliae*, *R. monacensis*, *R. felis*, *R. sibirica*, *R. slovaca*, *Ca.* R. tarasevichiae and *Ca.* R. jingxinensis, have been identified in ticks, animal hosts and humans in mainland of China [12–18]. In addition, several potential novel uncultured *Rickettsia* species, such as *Ca. R. hebeiii*, *Ca. R. tibetani*, *Ca. R. gannanii* and *R. subsp.* XY99, were also been reported in different areas of China according to the phylogenetic analysis of target gene loci [8, 19–21]. More importantly, *R. heilongjiangensis*, *R. raoultii*, *R. sibirica*, *R. subsp.* XY99, and *Ca.* R. tarasevichiae have been confirmed as the causative agents of human rickettsioses in mainland of China, and most of the human cases were mainly come from northeastern China, especially from Heilongjiang province [14, 22].

As in many other countries, dog has become a bonded family member. The dog population in China was estimated to be between 150 and 200 million in 2012 according to the records of the Chinese Center for Disease Control and Prevention [23], and the number of pet dogs is likely to have increased with the economic development and the improved living standards in urban populations. Dogs are important companion animals that sharing the environment within households even in the modern and urbanized society. Regardless the benefits of having pet dogs, dogs are reservoirs of zoonotic agents, serving as a nutrition source to many arthropods that also feed on humans, increasing the risk of zoonotic infections [24, 25]. Thus, dogs gain increasing public attention as they are considered as sentinels for infections by agents transmitted by vectors and may contribute to the spread of vector-borne diseases, including rickettsioses [26, 27]. However, to the best of our knowledge, only a few reports about the *Rickettsia* spp. infection in pets were available in China [18, 28, 29]. Currently, the status of *Rickettsia* spp. infection in pets remains poorly studied.

Harbin city, the capital city of Heilongjiang province, is the largest city located in northeastern of China. In this area, human rickettsiosis cases have been frequently reported in the recent years [6, 7, 30, 31]. Pet dogs play an important role in local community life. However, there is no epidemiological data about the *Rickettsia* spp. infection in pet dogs in urban areas of Harbin. Given the close bond that exists between humans and pet dogs, it is therefore prudent to better understand the public health risks that may be associated with the human-animal bond in local communities. To this end, we sought to obtain the information regarding *Rickettsia* species infecting pet dogs, investigate the epidemiology of rickettsial agents in pet dogs in urban areas of Harbin to better assess the risk of rickettsial infection in the local populations.

## Results

### Samples collection

**Fig. 1:**
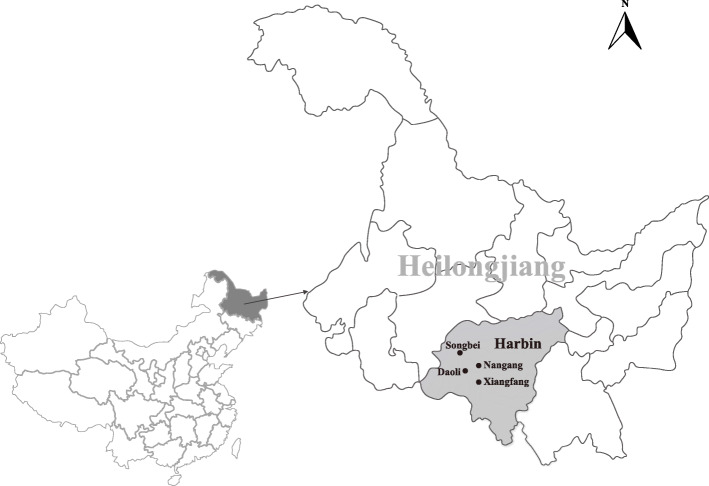
Map with the location of collecting sites of blood and infesting tick samples from pet dogs (●) in Harbin, China. It was drawn by us specific for this study, and plotted by combination of Surfer software version 4 (Golden Software, USA) and Photoshop CS 8.0.1 (Adobe Systems, USA)

During May to December of 2019, a total of 496 blood samples were collected from pet dogs, which were sent for vaccination, or for general inspection, or for veterinary assistance with some disease in local animal hospitals located in Songbei, Daoli, Nangang, and Xiangfang districts of Harbin city (Fig. 1). The detailed information, including gender, breed, age and number of these pet dogs are described in Table 1. The age of these pet dogs was between 5 months and 8 years. Across all the sampling sites, the percentage of the female and male pet dogs was 57.7 % (286/496) and 42.3 % (210/496), respectively. Meanwhile, 185 (37.3 %; 95 % CI: 33.0 %-41.6 %) of the 496 pet dogs examined had ticks that were collected, and a total of 343 ticks were collected. The most infested pet dog had 25 ticks at the time of collection. After the morphology identification and molecular confirmation based on the *rrs* gene of tick, all the ticks collected from pet dogs were identified as *Ixodes persulcatus* (74.6 %; 256/343), *Haemaphysalis concinna* (23.9 %; 82/343), and *Dermacentor silvarum* (1.5 %; 5/343).

**Table 1:** Prevalence of *Rickettsia* detected in blood and tick samples collected from pet dogs in Harbin, China

Sample type	Species	No. tested (n)	No. positive (n / %)	*Rickettsia* species (n / %)		
				*R. raoultii*	*Ca.* R. tarasevichiae	*R. felis*
**Blood**	Poodle	124	11 / 8.9	7 / 5.6	1 / 0.8	3 / 2.4
	Border Collie	103	6 / 5.8	5 / 4.9	0	1 / 1.0
	Siberian Husky	87	5 / 5.7	5 / 5.7	0	0
	Corgi	56	6 / 10.7	3 / 5.4	0	3 / 5.4
	Bichon Frise	48	4 / 8.3	3 / 6.3	0	1 / 2.1
	Labrador	43	3 / 6.9	3 / 7.0	0	0
	Chihuahua	35	5 / 14.3	2 / 5.7	1 / 2.9	2 / 5.7
	**Total**	**496**	**40 / 8.0**	**28 / 5.6**	**2 / 0.4**	**10 / 2.0**
**Tick**	*I. persulcatus*	256	35 / 13.7	25 / 9.8	10 / 4.0	0
	*H. concinna*	82	12 / 14.6	12 / 14.6	0	0
	*D. silvarum*	5	1 / 20.0	1 / 20.0	0	0
	**Total**	**343**	**48 / 14.0**	**38 / 11.1**	**10 / 2.9**	**0**

### Detection of Rickettsiae by PCR

The nested-PCR showed that 8.1 % (40/496, 95 % CI: 5.7 %-10.5 %) of the total DNAs extracted from blood samples were positive for both the *Rickettsia*-specific *gltA* and *17-kDa* genes (Table 1). The positive rate of *Rickettsia* in female pet dogs (9.1 %, 26/286, 95 % CI: 5.8 %-12.4 %) were higher than that in male pet dogs (6.7 %, 14/210, 95 % CI: 3.3 %-10.1 %), while the difference was not significantly (χ^2^ = 0.960, *df* = 1, *P* = 0.405) (Table 2). Meanwhile, no significantly difference was observed among the different breed of pet dogs (χ^2^ = 3.866, *df* = 6, *P* = 0.695) (Table 2). Notably, the positive rate in juveniles were significantly higher than that in adults (χ^2^ = 4.026, *df* = 1, *P* = 0.031) (Table 2). Of the 343 total DNAs extracted from ticks, 14.0 % (48/343, 95 % CI: 10.3 %-17.7 %) of the DNA samples were determined as positive for *Rickettsia* infection (Table 1). The positive rate of *Rickettsia* infection in ticks collected from pet dogs was significantly higher than that in pet dogs’ blood samples (χ^2^ = 7.594, *df* = 1, *P* = 0.004).

**Table 2:** Prevalence of *Rickettsia* detected in blood samples from pet dogs in Harbin, China

Parameters	No. tested (n / %)	No. positive (n / %)	χ^2^-value	*P*-value
**Gender**			0.960	0.405
Female	286 **/** 57.5	26 **/** 9.1		
Male	210 **/** 42.3	14 **/** 6.7		
**Breed**			3.866	0.695
Poodle	124 **/**	11 / 8.9		
Border Collie	103 **/**	6 / 5.8		
Siberian Husky	87 **/**	5 / 5.7		
Corgi	56 **/**	6 / 10.7		
Bichon Frise	48 **/**	4 / 8.3		
Labrador	43 **/**	3 / 6.9		
Chihuahua	35 **/**	5 / 14.3		
**Age**			4.026	0.031
Juvenile (< 1 year)	285 **/** 57.5	29 **/** 10.2		
Adult (≥ 1 year)	211 **/** 42.5	11 **/** 5.2		

Sequencing and further blast analysis of rickettsial *rrs*, *gltA* (1200 bp), *groEL* and *ompA* genes showed that three species of *Rickettsia*, including *R. raoultii* (70.0 %, 28/40), *Ca.* R. tarasevichiae (5.0 %, 2/40), and *R. felis* (25.0 %, 10/40), were identified from the 40 rickettsial *gltA* (720 bp) / *17-kDa* gene positive blood samples (Table 1). No co-infection of these *Rickettsia* species was detected in a single blood sample. *R. raoultii* displayed an extensive distribution in all breeds of pet dogs, and a significantly higher prevalence in pet dogs than other *Rickettsia* species (χ^2^ = 27.335, *df* = 2, *P* = 0.000).

In addition, only two species of *Rickettsia*, *R. raoultii* (79.2 %, 38/48) and *Ca.* R. tarasevichiae (20.8 %, 10/48), were identified from all the 48 positive tick samples. Specifically, *R. raoultii* was identified in the ticks of *I. persulcatus* (9.8 %, 25/256), *H. concinna* (14.6 %, 12/82) and *D. silvarum* (20.0 %, 1/5) with an overall prevalence of 11.1 % (38/343, 95 % CI: 7.8 %-14.4 %), while *Ca.* R. tarasevichiae was identified in the ticks of *I. persulcatus* alone, with an overall prevalence of 2.9 % (10/343, 95 % CI: 1.1 %-4.7 %), which was significantly lower than that of *R. raoultii* in ticks (χ^2^ = 17.562, *df* = 1, *P* = 0.000).

### Genetic and phylogenetic analysis

The nearly complete *rrs* gene, *gltA* gene, *groEL* gene, as well as the partial *ompA* gene of rickettsial were also amplified from the positive total DNA samples screened by nested-PCR targeted *gltA* and *17-kDa* genes. Almost full-length *rrs* gene sequences (n = 88), the *gltA* gene sequences (n = 88), the *groEL* gene sequences (n = 80), and the *ompA* gene sequences (n = 83) were obtained from the positive DNA samples.

**Fig. 2:**
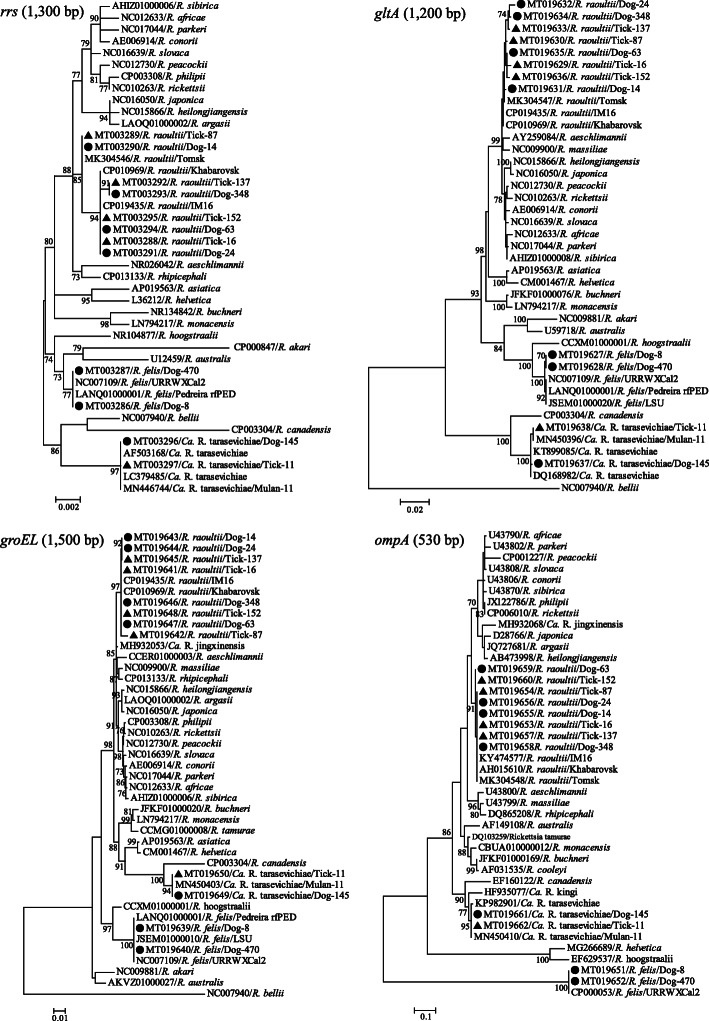
Molecular identification of *Rickettsia* spp. detected in the present study based on the phylogenetic trees of *rrs*, *gltA*, *groEL* and *ompA* genes. The trees were reconstructed based on the maximum likelihood method implemented in PhyML v3.0, and mid-point rooted for clarity and the scale bar represents the number of nucleotide substitutions per site. Bootstrap values were calculated with 1000 replicates of the alignment. Sequences marked with black solid triangle (▲) and black dot (●) indicate the representative sequences determined from blood samples and tick samples, respectively

Sequence blast analysis showed that the nucleotide similarity of the *rrs*, *gltA*, *groEL*, and *ompA* genes that we obtained and the corresponding sequences of *R. raoultii* available in GenBank were 99.7 %-100 %, 99.6 %-100 %, 99.4 %-100 %, and 99.6 %-100 %, respectively. In the phylogenetic trees based on *rrs*, *gltA*, *groEL* and *ompA* gene, which were reconstructed based on GTR + Γ + I model using the maximum-likelihood (ML) implemented in PhyML v3.0, the representative sequences of *R. raoultii* recovered in blood and tick samples were clutered together with the corresponding sequences of *R. raoultii* available in GenBank, and shared close genetic relationship with strains of *R. raoultii* isolate Tomsk, *R. raoultii* strain IM6, and *R. raoultii* strain Khabarovsk (Fig. 2).

The sequences of *Ca.* R. tarasevichiae obtained in this study were closely related to each other, with 99.8–100 % nucleotide similarity. The representative sequences of *rrs*, *gltA*, and *ompA* gene of *Ca.* R. tarasevichiae that we obtained (Dog-145 and Tick-11) shared 99.7 %, 100 % and 99.4 % of nucleotide similarity with the corresponding sequences of *Ca.* R. tarasevichiae strains available in GenBank, respectively; while the *groEL* gene of *Ca.* R. tarasevichiae that we obtained shared 100 % nucleotide similarity with the *Ca.* R. tarasevichiae strain Mulan-11, which was identified in ticks collected in the remote areas of Harbin in our previous study (in press). Consistent with the results of genetic comparison, Dog-145 and Tick-11 were clustered together with other *Ca.* R. tarasevichiae strains available in GenBank in all four phylogenetic trees (Fig. 2).

In the sequence blast analysis, the *R. felis* detected in this study presented the highest nucleotide similarity (100 %, 99.8 %, 100 %, and 100 %) with *R. felis* URRWXCal2 for *rrs*, *gltA*, *groEL*, and *ompA* genes, respectively. In the phylogenetic trees, the representative sequence of *R. felis* that we identified was clustered with *R. felis* isolates, and showed the closet genetic relationship with *R. felis* URRWXCal2.

## Discussion

Investigation of neglected zoonotic pathogens in domestic pets and vectors infecting them is important in the prevention and control of zoonotic diseases [32]. In the present study, blood samples of pet dogs, and tick samples infested in these pet dogs, were collected and screened for rickettsial agents in urban areas of Harbin city, northeastern China. Three rickettsiae species (*R. raoultii*, *Ca.* R. tarasevichiae and *R. felis*) and two rickettsiae species (*R. raoultii* and *Ca.* R. tarasevichiae), were identified from pet dogs’ blood samples and the infested tick samples, respectively. Additionally, the overall positive rate of *Rickettsia* infection in ticks (14.0 %) was significantly higher than that in pet dogs’ blood samples (8.1 %). Moreover, the overall positive rate of *Rickettsia* infection shown no correlation with gender or breed of pet dogs, but significantly associated with age (positive rate in juveniles were significantly higher than that in adults), which might because the underdeveloped immune system of juveniles caused the slowly recovery from *Rickettsia* infection; thus, the examined juvenile dogs were in acute phase of infection at the time of blood collection, and they were easier to identified. It might also because the juvenile individuals are more active than adult individuals, more frequently to contact with outdoors, and also have more chance to interact with other dogs. More importantly, *R. raoultii*, *Ca.* R. tarasevichiae and *R. felis*, which was identified in the present study, have been confirmed as the causative agents of rickettsioses in humans [7, 33–37]. Given that pet dogs are considered as human companions and share the household environment with humans, and they have been identified as a notable mammalian reservoir hosts for many *Rickettsia* species and may play an important role as source of human infection, it is meaningful and necessary that actively surveillance of rickettsial bacteria carried by pet dogs for better assess the risk of rickettsial infection in the local populations.

*Rickettsia raoultii* was first identified in ticks collected from Russia in 1999 [38], and it has been detected in at least 12 tick species collected from a large geographical spread in China [14, 15, 19, 30, 39], which suggest the wide geographical and host species distribution of *R. raoultii* in China. In this study, the positive rate of *R. raoultii* detected in blood samples (5.6 %) or in tick samples (11.1 %) were significantly higher than other species of *Rickettsia* identified in the present study, suggested that *R. raoultii* was the most predominate *Rickettsia* species infecting pet dogs in this area. In addition, *R. raoultii* was identified in seven species of pet dogs and three species of ticks, and no significantly difference was observed among the different breed of pet dogs. Although only one *R. raoultii* positive sample was detected from *D. silvarum*, the positive rate of *R. raoultii* detected in *D. silvarum* was significantly higher than that in *H. concinna* or in *I. persulcatus*. Our results are consistent with previous reports that *Dermacentor* spp., was the major vector for *R. raoultii* [14, 40]. Moreover, the positive rate of *R. raoultii* detected in *I. persulcatus* was 9.8 %, which was lower than that in *H. concinna*, or in *D. silvarum*. However, to our knowledge, this is the first report about *R. raoultii* identified from *I. persulcatus* in Harbin, although *I. persulcatus* have been confirmed as the predominant tick species in Heilongjiang province [14].

*Candidatus* Rickettsia tarasevichiae is an emerging tick-borne rickettsiae species initially found in the ticks of *I*. *persulcatus* in Russia [41], it’s also has been confirmed pathogenic to human, and the first human case was identified in China in 2013 [7, 42]. In the present study, *Ca.* R. tarasevichiae was exclusively detected in *I*. *persulcatus* out of three tick species collected in this study, which are consistent with previous reports presented that *Ca.* R. tarasevichiae was closely associated with *I*. *persulcatus* [14, 43]. Moreover, *I*. *persulcatus* is a common human parasite in northeastern of China, and it is also known to transmit a variety of other human pathogens, such as *Borrelia burgdorferi*, *Babesia* spp., *Anaplasma phagocytophilum* [22]. These suggest that *I*. *persulcatus* could contribute greatly to the spread of *Ca.* R. tarasevichiae in China, and also raised the possibility of co-infection of humans by *Ca.* R. tarasevichiae and other tick-borne pathogens. Additionally, we did not amplify DNA of *Ca.* R. tarasevichiae from *H*. *concinna* collected in this study, although it has been confirmed to be the vector for *Ca.* R. tarasevichiae in northeastern China previously [14, 22].

*Rickettsia felis* also is an emerging vector-borne rickettsiae species distributed throughout the world [44–47], and the biological vector is the cat flea, *Ctenocephalides felis* [48], although it has also been found in other arthropods (ticks, mites, booklice and mosquitoes). It has been confirmed as the causative agent of flea-borne spotted fever (FBSF) in humans, and posing a global emerging threat to human health [49, 50]. Meanwhile, domestic dogs are considered mammalian reservoir hosts for *R. felis* [48]. In the present study, *R. felis* was detected from blood samples of pet dogs with an overall prevalence of 2.0 %. Previous study has showed a high seroprevalence (47 %), and a low PCR-based positive rate (0.8 %) of *R. felis* infection in dogs from China [18]. The positive rate of *R. felis* infection in dogs based on PCR detection was significantly lower than that determined by serology assay, might attribute to the fact that there is at least some serological cross reactivity between *R. felis* and other *Rickettsia* spp. present in China [51]. Additionally, 10.3 % of the ticks collected from dogs in previous study were PCR positive, while no positive sample was determined in tick samples in this study. The incongruent results might because the tick species collected in previous study was *Rhipicephalus sanguineus*, which also have been confirmed as the vector for *R. felis* [52], no reports about the *R. felis* infection in the tick species collected in present study are available.

## Conclusions

In summary, three rickettisae species pathogenic to humans (*R. raoultii*, *Ca.* R. tarasevichiae, and *R. felis*) were identified from pet dogs’ blood and the infested ticks in urban areas of Harbin city. In the light of the co-existence of humans and pet dogs, these results indicate the potential transmission risk of human rickettisal infections from pet dogs through ectoparasites, and also remind the public that reduce the contact of pet dogs with outdoors, and periodically remove the ectoparasites, will help to prevent the rickettisal infection of pet dogs and humans. From the “One health” perspective, these results also suggest that sharing information between public and animal health on ectoparasites of companion animals and the disease agents they carry, could contribute to surveillance of rickettsioses in human in China.

## Methods

### Collection of blood and tick samples

During May to December of 2019, blood and tick samples were randomly collected from pet dogs, which were sent for vaccination, or for general inspection, or for veterinary assistance with some disease in animal hospitals located in urban areas (Songbei, Daoli, Nangang, and Xiangfang districts) of Harbin city, Heilongjiang province of China. The blood samples were collected into vacutainer tubes containing ethylenediaminetetraacetic acid (EDTA) anticoagulant, and the adult ticks were collected from the animal body surface by hand searching. Species identification of ticks were firstly determined according to their morphology as previously described [53, 54], then further confirmed by amplifying, sequencing and analyzing the 16 S ribosomal RNA (*rrs*) gene of ticks [55]. The detailed information of pet dogs was recorded by veterinarians before blood sample collection.

### DNA extraction and***Rickettsiae***detection

Total DNA was extracted from 200 µL of whole blood samples by using the Blood DNA Extraction Kit (OMEGA, USA) according to the manufacturer’s instructions. The ticks were soaked in 70 % ethanol for sterilisation and then washed three times with double distilled water (ddH_2_O). The whole body of each tick was homogenized, and the total DNA was extracted from individual tick by using a Tissue DNA Extraction Kit (Omega, USA) according to the manufacturer’s protocol. The extracted DNA was eluted in 80 µL autoclaved double distilled water (ddH_2_O) and stored at -20˚C.

Using the extracted DNA as templates, the rickettsial DNA was detected by nested-PCR with the primers targeting a 720-bp citrate synthase encoding gene (*gltA*) and a 450-bp 17 kilodalton antigen encoding gene (*17-kDa*) segments as previously described [56, 57]. During the detection of *Rickettsiae*, a DNA sample of *R. japonica* and ddH_2_O were used as positive and negative control, respectively. To prevent contamination, the template isolation, PCR mix preparation, template DNA addition, and agarose gel electrophoresis were performed in separate rooms, and dedicated pipets and tips with filter elements inside were used.

### Amplification of rickettsial***rrs***, ***gltA***, ***groEL***, and***ompA***genes

Only both the *gltA* and *17-kDa* gene positive total DNA samples were selected to amplify the nearly entire *rrs* gene (1300 bp), *gltA* gene (1200 bp), heat shock protein-60 encoding gene (*groEL*, 1500 bp) as well as the partial outer membrane protein A encoding gene (*ompA*, 530 bp) of rickettsial to determine the species of *Rickettsia* detected. All the primer sequences and PCR parameters used in this study were shown in Table 3 detailed.

**Table 3:** Primers used in this study

Gene	Primer	Sequence (5’→3’)	Amplicon (bp)	Reference
Tick *rrs*	16SF	F: GTATTTTGACTATACAAAGGTATTG	300	[55]
	16SR	R: TATTACGCTGTTATCCCTAGAGTATT		
*gltA*	Ric-CS409d	F: CCTATGGCTATTATGCTTGC	720	[57]
	Ric-CS535d	F: GCAATGTCTTATAAATATTC		
	Ric-CS1258n	R: ATTGCAAAAAGTACAGTGAACA		
	Ric-CS2d	F: ATGACCAATGAAAATAATAAT	1200	[7]
	Ric-CSEndr	R: CTTATACTCTCTATGTACA		
*17-kDa*	Rr17k.1p	F: TTTACAAAATTCTAAAAACCAT	450	[56]
	Rr17k.90p	F: GCTCTTGCAACTTCTATGTT		
	Rr17k.539n	R: TCAATTCACAACTTGCCATT		
Rickettsial *rrs*	Ric-16SF	F: GAACGAACGCTATCGGTATGC	1300	This study
	Ric-16SR1	R: AATTTTACCGTGGTTGGCTGC		
	Ric-16SR2	R: TGCCTCTTGCGTTAGCTCAC		
*groEL*	Ric-ESL-F1	F: GGTAAATGGGCAGGYACCGAA	1500	This study
	Ric-ESL-R1	R: GAAGCAACRGAAGCAGCATCTTG		
	Ric-ESL-F2	F: ATCGTTATGAAAGAAAGCGAYG	1500	This study
	Ric-ESL-R2	R: AGWGCAGTACGCACTACTTTAGC		
*ompA*	Rr190k.71p	F: TGGCGAATATTTCTCCAAAA	530	[56]
	Rr190k.602n	R: AGTGCAGCATTCGCTCCCCCT		
	Rr190k.720n	R: TGCATTTGTATTACCTATTGT		

*F* Forward primer. *R* Reverse primer. Y = C or T. W = A or T

### Sequencing of the PCR products

The PCR products were examined in agarose gel using an Image Analyzer (Bio-Rad, USA) after electrophoresis. PCR products less than 800 bp were sequenced directly, and the PCR products more than 800 bp were firstly purified using a Gel Extraction Kit (Qiagen, USA) according to the manufacturers’ protocols, then cloned into pMD19-T plasmid (TaKaRa, China) and transformed into *E. coli* JM109 competent cells. For each amplicon, the positive inserts were confirmed by PCR, and three positive clones were sequenced with the universal M13 forward and reverse primers in cloning vector. All the sequencing procedures were performed by Sangon Biotechnology Company in China.

### Sequence comparison and phylogenetic analysis

The obtained nucleotide sequences from the target genes of rickettsiae were edited and assembled using SeqMan program (DNASTAR, Madison, WI). Multiple nucleotide sequence alignment was performed using MUSCLE (default parameters) method in the MEGA program, version 6.0 [58]. The nucleotide identities were calculated using the DNASTAR Lasergene 12 Core Suite [59]. All the sequences obtained in this study were compared with the corresponding sequences available in GenBank using BLAST software, and the sequence data have been submitted to GenBank databases under accession numbers MT003286-MT003297 and MT019627-MT019662.

The best-fit nucleotide substitution models for phylogenetic analysis based on the different genes of rickettsiae were determined using jModel Test [60]. Phylogenetic trees were reconstructed using Maximum likelihood (ML) method implemented in PhyML v3.0 software [61]. The reliability of branches in the inferred trees was evaluated by bootstrap analysis of 1000 replicates, and the values over 70 % were considered as significant differences for presentation. All phylogenetic trees were mid-point rooted for purposes of clarity only.

### Statistical data analysis

Statistical analysis of the obtained data was performed using Statistical Package for Social Sciences Version 21.0 software (SPSS, Chicago, IL, USA). Chi-square test or Fisher’s exact test was used for calculating the *P*-value to determine the differences of the positive rate of rickettsiae. Statistical significance was defined as *P* < 0.05.

## Data Availability

The data that support the findings of this study are openly available in GenBank at , under the accession numbers as MT003286-MT003297 and MT019627-MT019662.

## Abbreviations

*R. raoultii*: *Rickettsia raoultii*
*R. heilongjiangensis*: *Rickettsia heilongjiangensis*
*R. rickettsia*: Rickettsia Rickettsia
*R. conorii*: *Rickettsia conorii*
*R. aeschlimannii*: *Rickettsia aeschlimannii*
R. massiliae: *Rickettsia massiliae*
*R. monacensis*: *Rickettsia monacensis*
*R. felis*: Rickettsia felis
*R. sibirica*: Rickettsia sibirica
*R. slovaca*: *Rickettsia slovaca*
*Ca.* R. tarasevichiae: *Candidatus* Rickettsia tarasevichiae
*Ca.* R. jingxinensis: *Candidatus* Rickettsia jingxinensis
